# Family functioning and psychological well-being in Chinese early adolescents: a three-wave study of future-oriented coping and gender differences

**DOI:** 10.3389/fpsyg.2026.1945147

**Published:** 2026-09-22

**Authors:** Qiangqiang Ma, Qianwen He, Xinhui Li, Hongyu Zhou

**Affiliations:** School of Marxism, Jilin University of Finance and Economics, Jilin, China

**Keywords:** Chinese adolescents, early adolescence, family functioning, future-oriented coping, gender differences, psychological well-being

## Abstract

**Introduction:**

Adolescent psychological well-being has been linked to family functioning, yet the longitudinal processes underlying this association remain insufficiently understood. This study examined whether future-oriented coping mediates the prospective association between family functioning and psychological well-being and whether this indirect association varies by gender.

**Methods:**

A three-wave longitudinal study was conducted among 1,500 early adolescents from eight public secondary schools in Changchun, China. Family functioning was assessed at time 1, future-oriented coping at time 2, and psychological well-being at all three waves. Structural equation modeling and multi-group analyses were used to examine the hypothesized mediation model and gender differences while accounting for prior psychological well-being and demographic covariates.

**Results:**

Higher family functioning was prospectively associated with greater future-oriented coping and higher psychological well-being. Future-oriented coping partially accounted for the longitudinal association between family functioning and psychological well-being (indirect effect: *β* = 0.195, 95% CI [0.132, 0.258]), and this indirect association was stronger among girls than boys (difference in indirect effects: *β* = 0.191, 95% CI [0.054, 0.329]).

**Discussion:**

These findings suggest that future-oriented coping may represent one potential temporally ordered pathway linking family functioning to later psychological well-being, with the strength of this pathway differing between girls and boys.

## 1 Introduction

Adolescence is a developmental period characterized by rapid physical, social, emotional, and cognitive changes that play a fundamental role in shaping future health and development (Verzeletti et al., 2016). During this stage, young people strive to develop their personal and social identities while simultaneously facing multiple challenges, including family and peer pressures as well as broader societal expectations (Yousefi Afrashteh and Hasani, 2022), all of which may influence their overall well-being. Psychological well-being, as conceptualized by Ryff and Singer (1996), reflects positive psychological functioning across dimensions such as autonomy, environmental mastery, personal growth, positive relationships, purpose in life, and self-acceptance. Optimal psychological well-being during adolescence has been linked to both immediate life satisfaction and long-term developmental outcomes (Kansky et al., 2016). Therefore, understanding the factors associated with psychological well-being during adolescence appears particularly important.

Coping is a dynamic process through which individuals use cognitive, emotional, and behavioral strategies to manage stressful or challenging situations and their potential effects on development and well-being (Gervais and Jose, 2020). Future-oriented coping represents a distinct perspective within coping research because it emphasizes anticipatory cognitive and behavioral efforts directed toward future demands rather than only responses to stressors that have already occurred. It comprises two complementary forms: preventive coping and proactive coping (Gan et al., 2007). Preventive coping involves preparing for uncertain future threats by accumulating resources to reduce potential stress, whereas proactive coping emphasizes anticipated challenges, opportunities, and goal attainment through planning and resource building (Gan et al., 2007; Serrano et al., 2021). Thus, future-oriented coping encompasses both preparation for potential future difficulties and proactive efforts toward future goals.

During adolescence, future-oriented coping may be especially relevant because young people increasingly encounter academic, interpersonal, and developmental demands that require planning, self-regulation, and preparation for future transitions. By anticipating demands and building coping resources before difficulties fully unfold, future-oriented coping may support adolescents’ preparedness for future challenges and may be associated with better subsequent psychological adjustment. Consistent with this possibility, both proactive and preventive coping have been associated with greater subjective well-being and lower depression among adolescents, although associations with anxiety may differ between the two components (Serrano et al., 2021). However, the prospective role of overall future-oriented coping in adolescent psychological well-being remains underexamined.

Family functioning has long been regarded as a key factor in adolescent development. According to the McMaster model, family functioning refers to the family system’s ability to support the physical, emotional, and social well-being of its members through effective problem-solving, communication, role distribution, emotional responsiveness, affective involvement, and behavior control (Miller et al., 2000). Collectively, these dimensions reflect how families manage everyday functioning and respond to developmental and environmental demands.

Much of the existing evidence on family functioning and adolescent psychological adjustment has been derived from Western populations. However, the meaning and expression of family functioning may vary across sociocultural contexts (Shek and Ma, 2010). In China, the family occupies a central role in adolescent development and serves as a primary source of emotional support, socialization, and behavioral guidance (Xiang Li et al., 2024). Consequently, examining the association between family functioning and adolescent psychological well-being within the Chinese sociocultural context is particularly relevant. Future-oriented processes also appear relevant in Chinese adolescent samples. A five-wave study found that future time perspective was prospectively associated with life satisfaction among Chinese adolescents (Xiaobao Li et al., 2023), while research with Chinese high school students has linked parental autonomy support with stronger future-oriented coping (Zeng et al., 2022). Chinese cultural traditions also emphasize family harmony, interdependence, and parental authority as foundations for both individual and collective functioning (Shek, 2005). At the same time, rapid socioeconomic and cultural changes in contemporary China have transformed family dynamics and adolescents’ developmental experiences, highlighting the importance of examining family functioning within this evolving sociocultural environment (Xia et al., 2004; Xu and Xia, 2014).

Family functioning has been linked to adolescents’ coping processes across a range of studies. Adolescents reporting more positive family relationships tend to use more active coping, whereas conflictual or poorly communicative family environments have been associated with greater reliance on avoidant responses (Wadsworth, 2015; Cicognani, 2011; Zimmer-Gembeck and Locke, 2007). More specifically, supportive family relationships may provide involvement, structure, and autonomy support that help adolescents develop active coping resources and prepare for anticipated demands (Zimmer-Gembeck and Locke, 2007). Consistent with this possibility in a Chinese context, parental autonomy support has been positively associated with future-oriented coping among Chinese high school students (Zeng et al., 2022). Taken together, these findings suggest that more positive family functioning may be associated with stronger future-oriented coping in adolescents.

Growing evidence further suggests that coping processes may help explain how family functioning becomes associated with adolescent psychological adjustment. For example, adolescents with lower levels of planned coping or greater dependence on avoidant strategies often report higher stress and poorer adjustment outcomes, whereas adolescents with stronger internal coping resources tend to demonstrate greater resilience (Hickey et al., 2017). Maladaptive coping patterns such as rumination have been associated with internalizing symptoms among adolescents from dysfunctional family environments, whereas support-seeking and adaptive coping appear to serve protective roles (Francisco et al., 2016). Similarly, family connectedness has been linked to healthier emotional regulation and improved psychological outcomes through its influence on coping capacities (Gervais and Jose, 2020). Although prior studies suggest that coping may help explain the association between family functioning and adolescent adjustment, relatively little attention has been given to future-oriented coping specifically.

Several empirical findings provide preliminary support for this proposed mediational pathway. Rodriguez et al. (2014) found that adolescents from poorly functioning families reported lower use of active coping strategies and higher levels of emotional and behavioral difficulties, with coping processes partially accounting for these associations. Similarly, Shao et al. (2022) reported that family functioning positively predicted resilience among Chinese adolescents both directly and indirectly through positive coping. In addition, adolescents’ future orientation has been linked to better psychological well-being over time through more adaptive coping processes (Chua et al., 2015). Collectively, these findings suggest meaningful connections among family functioning, coping processes, and psychological well-being, supporting the possibility that future-oriented coping may mediate the association between family functioning and adolescent well-being. Nevertheless, existing evidence provides limited insight into the temporal ordering among family functioning, future-oriented coping, and psychological well-being because much of the literature has relied on cross-sectional designs. Prospective research is therefore needed to examine whether future-oriented coping represents a temporally ordered pathway between family functioning and later psychological well-being. Consequently, prospective longitudinal research is needed to examine whether family functioning is associated with subsequent psychological well-being through future-oriented coping over time.

From a theoretical perspective, family systems theory proposes that healthier family functioning contributes to better psychological adjustment among family members (Baptist and Hamon, 2022; Persram et al., 2025). Ecological systems perspectives similarly emphasize the central role of family environments in shaping adolescents’ developmental capacities and coping resources (Bronfenbrenner, 1979; Zeng et al., 2022). In parallel, Lazarus and Folkman’s transactional model of stress and coping suggests that coping strategies mediate the effects of environmental conditions and stressors on psychological outcomes (Lazarus and Folkman, 1984; Yang et al., 2022; Acoba, 2024). Together, these frameworks imply that well-functioning families may provide adolescents with emotional security, interpersonal support, and opportunities to develop planning and coping resources that facilitate future-oriented coping with anticipated challenges. In turn, such anticipatory coping may provide one pathway through which family functioning is associated with later psychological well-being.

In addition to mediating processes, emerging evidence suggests that gender may moderate the associations among family functioning, coping strategies, and psychological outcomes during adolescence. Developmental and socialization perspectives propose that boys and girls are exposed to different emotional expectations and coping socialization experiences, which may influence both the use and effectiveness of coping strategies. Gender socialization theories further suggest that these experiences may shape not only adolescents’ coping resources but also the extent to which family environments influence psychological adjustment (Rose and Rudolph, 2006). Consequently, associations among family functioning, future-oriented coping, and psychological well-being may vary across gender groups. Empirical findings indicate that gender differences in coping emerge during adolescence, although observed patterns vary across studies and cultural settings. For instance, girls have often been found to report greater use of emotion-focused, support-seeking, and interpersonal coping strategies, whereas boys may be more likely to engage in disengagement or avoidant coping in certain contexts (Perzow et al., 2021). Longitudinal evidence further suggests that girls frequently report higher engagement in active coping and support-seeking behaviors during adolescence (Flannery et al., 2018).

Gender differences have also been observed in the associations between coping strategies and psychological adjustment outcomes, suggesting that coping processes may partly contribute to gender differences in adolescent well-being (Wilson et al., 2005; Choi et al., 2026; Malooly et al., 2017). Extending this perspective, it is plausible that gender may condition the strength of associations among family functioning, future-oriented coping, and psychological well-being. Such moderating processes may be particularly relevant during early adolescence (approximately 11–14 years), a developmental period characterized by heightened sensitivity to interpersonal relationships and increasingly salient gender-role expectations. To our knowledge, relatively few studies have examined whether gender conditions the longitudinal pathways linking family functioning, future-oriented coping, and psychological well-being, particularly among adolescents in non-Western cultural contexts.

Using a sample of Chinese early adolescents, the present three-wave prospective study examined whether future-oriented coping at T2 mediated the longitudinal association between family functioning at T1 and psychological well-being at T3 after accounting for prior levels of psychological well-being, and whether these pathways differed across gender. The following hypotheses were proposed (see Figure 1):

**Figure 1:**
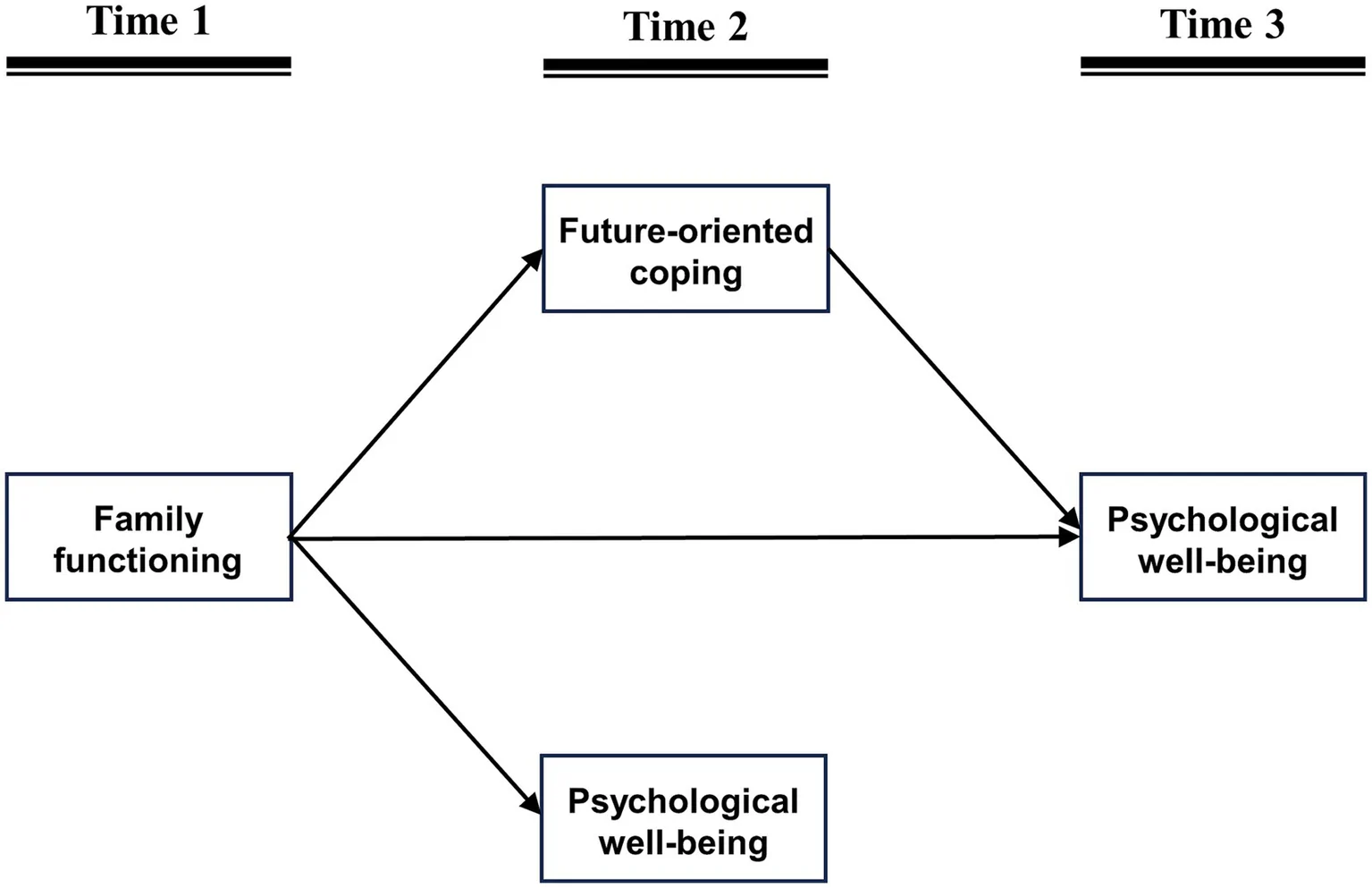
Hypothesized longitudinal mediation model.

*H1*: Family functioning at T1 would positively predict subsequent psychological well-being at T2 and T3 after controlling for prior levels of psychological well-being.

*H2*: Family functioning at T1 would positively predict future-oriented coping at T2.

*H3*: Future-oriented coping at T2 would positively predict psychological well-being at T3 after controlling for prior psychological well-being.

*H4*: Future-oriented coping at T2 would mediate the longitudinal association between family functioning at T1 and psychological well-being at T3.

*H5a*: At least one of the longitudinal structural associations among family functioning, future-oriented coping, and psychological well-being would differ across gender groups.

*H5b*: The indirect longitudinal association between family functioning and psychological well-being through future-oriented coping would vary across gender.

## 2 Materials and methods

The study was conducted in accordance with the Declaration of Helsinki, and approved by the Ethics Committee of Jilin University of Finance and Economics (approval no. J-YL.252037, June 26, 2025). Written informed consent was obtained from parents, and adolescents provided written assent before participation.

### 2.1 Participants

Participants were 1,500 early adolescents recruited from eight public secondary schools in Changchun, Jilin Province, China. At Time 1 (T1; September 2025), participants ranged in age from 12 to 14 years (M = 13.02, SD = 0.82). The sample included 799 girls (53.3%) and 701 boys (46.7%).

Regarding parental education, 30.6% of fathers had a middle school education or below, 34.5% had completed high school, 20.1% had completed college, and 14.9% held a bachelor’s degree or higher. Corresponding percentages for mothers were 35.9, 37.2, 14.7, and 12.1%, respectively. Regarding perceived family socioeconomic status (SES), 25.3% reported low SES, 55.1% reported middle SES, and 19.6% reported high SES.

### 2.2 Procedure

Data were collected across three waves separated by three-month intervals. Time 1 (T1) data were collected in September 2025, Time 2 (T2) data were collected in December 2025, and Time 3 (T3) data were collected in March 2026. Trained research assistants administered the questionnaires in Chinese in classroom settings during regular school hours using standardized instructions. Participants completed the questionnaires confidentially, and responses were linked across waves using nonidentifying participant codes. Questionnaire completion required approximately 30–60 min at each wave. No incentives or compensation were provided for participation.

At T1, 1,500 adolescents participated in the study. At T2, 1,458 participants remained (retention rate = 97.2%); 42 participants were not retained because of absence during data collection (*n* = 31) or the exclusion of invalid questionnaires (*n* = 11). At T3, 1,407 participants remained (retention rate = 93.8% relative to T1); an additional 51 participants were not retained because of absence during data collection (*n* = 39) or the exclusion of invalid questionnaires (*n* = 12). The overall attrition rate from T1 to T3 was 6.2%. Questionnaires were classified as invalid if more than 50% of the items on any of the three main study measures were unanswered or if they showed clearly non-attentive or mechanically patterned responding (e.g., extended uniform or repetitive response patterns or multiple responses to single-response items). The measurement schedule across the three waves is summarized in Table 1.

**Table 1:** Measurement schedule across the three study waves.

Variable	Time 1	Time 2	Time 3
Family functioning	✓	—	—
Future-oriented coping	—	✓	—
Psychological well-being	✓	✓	✓
Demographic variables	✓	—	—

### 2.3 Measures

#### 2.3.1 Family functioning

Family functioning was measured at T1 using the 33-item Chinese Family Assessment Instrument (C-FAI), which was originally developed and validated for Chinese populations (Shek, 2002). Subsequent psychometric evidence has further supported its use among Chinese adolescents in mainland China (Shek et al., 2023). The instrument comprises five dimensions: mutuality (12 items), communication (9 items), conflict and harmony (6 items), parental concern (3 items), and parental control (3 items). Responses were recorded on a 5-point Likert scale ranging from 1 (strongly disagree) to 5 (strongly agree). Subscale scores were calculated according to the instrument scoring procedures, and an overall family functioning score was computed by averaging the five subscale scores. Higher scores indicate better family functioning. In the present study, Cronbach’s α for the C-FAI was 0.88.

#### 2.3.2 Future-oriented coping

Future-oriented coping was measured at T2 using the Chinese-language 16-item Future-Oriented Coping Inventory (FCI), developed and evaluated in Chinese student samples by Gan et al. (2007) and subsequently used among Chinese adolescents (Zeng et al., 2022). The instrument comprises two 8-item dimensions: preventive coping and proactive coping. Responses were rated on a 5-point Likert scale ranging from 1 (strongly disagree) to 5 (strongly agree). Subscale scores were calculated according to the instrument scoring procedures, and an overall future-oriented coping score was computed by averaging the two subscale scores. Higher scores indicate stronger future-oriented coping tendencies. Cronbach’s α for the FCI was 0.83 in the present study.

#### 2.3.3 Psychological well-being

Psychological well-being was assessed at T1, T2, and T3 using an existing 24-item Chinese-language version of the Psychological Well-Being Scale (PWBS), adapted from Ryff (1989) original measure. This version has previously been used and psychometrically supported among Chinese adolescents in Hong Kong (Sun et al., 2016; Chan et al., 2022). The instrument comprises six dimensions, each represented by four items: autonomy, environmental mastery, personal growth, positive relations with others, purpose in life, and self-acceptance. Subscale scores were calculated according to the instrument scoring procedures, and an overall psychological well-being score was computed by averaging the six subscale scores. Higher scores indicate greater psychological well-being. Cronbach’s α values were 0.82, 0.85, and 0.84 at T1, T2, and T3, respectively.

#### 2.3.4 Demographic variables

Participants reported their age, gender, perceived family SES, and parental educational attainment at T1. Perceived family SES was assessed using a single self-report item asking adolescents to evaluate their family’s socioeconomic standing relative to other families and was categorized as low, middle, or high. Father’s and mother’s educational attainment were assessed separately using ordinal response categories reflecting increasing levels of educational attainment. Age, perceived family SES, and parental education were included as covariates in all structural models, whereas gender was examined as a grouping variable.

### 2.4 Statistical analysis

All statistical analyses were conducted using R version 4.5.3. Among the 1,407 participants retained through T3, 5.4% of item responses were missing across the three waves. Subscale scores were calculated when at least 75% of the constituent items had valid responses, using the mean of the available items. Otherwise, the corresponding subscale score was coded as missing. After applying this scoring rule, 2.8% of the resulting subscale-score observations entered as indicators in the SEM remained missing and were handled using full information maximum likelihood (FIML) estimation under the missing-at-random (MAR) assumption, consistent with a hybrid proration–FIML approach (Wu et al., 2022). Attrition analyses, descriptive statistics, and Pearson correlation coefficients were computed for all study variables.

Given the exclusive use of self-report measures, potential common method variance was evaluated separately at each measurement wave. At each wave, Harman’s single-factor test and a one-factor confirmatory factor analysis (CFA) were conducted using the item responses collected at that wave.

Structural equation modeling (SEM) was performed using the lavaan package in R to examine the hypothesized longitudinal mediation model. For the SEM analyses, the latent constructs were specified using the questionnaire subscale scores as indicators. Family functioning at T1 was specified as the predictor, future-oriented coping at T2 as the mediator, and psychological well-being at T3 as the outcome variable.

The model included the hypothesized prospective paths from family functioning at T1 to future-oriented coping at T2 and psychological well-being at T2 and T3, and from future-oriented coping at T2 to psychological well-being at T3. Two autoregressive paths (psychological well-being at T1 → psychological well-being at T2 and psychological well-being at T2 → psychological well-being at T3) were included to account for temporal stability in psychological well-being. The path from psychological well-being at T1 to future-oriented coping at T2 served as a baseline-adjustment path, allowing subsequent coping to be modeled while accounting for prior psychological functioning. The direct path from family functioning at T1 to psychological well-being at T3 allowed estimation of the prospective association remaining after accounting for the indirect pathway through future-oriented coping, thereby permitting partial rather than assuming full mediation. Covariances between family functioning and psychological well-being at T1 and between future-oriented coping and psychological well-being at T2 accounted for associations between constructs assessed at the same measurement occasion. Age, perceived family SES, father’s educational attainment, and mother’s educational attainment were included as demographic covariates in the structural models.

Model fit was evaluated using multiple goodness-of-fit indices, including the chi-square/degrees of freedom ratio (χ^2^/df < 3), comparative fit index (CFI ≥ 0.90), Tucker–Lewis index (TLI ≥ 0.90), root mean square error of approximation (RMSEA ≤ 0.06), and standardized root mean square residual (SRMR ≤ 0.06) (Schermelleh-Engel et al., 2003).

Direct and indirect effects were evaluated using bias-corrected bootstrap procedures with 5,000 resamples and 95% confidence intervals (CIs). Effects were considered statistically significant when the confidence interval did not include zero.

After establishing measurement invariance across gender, a multi-group SEM was estimated for girls and boys. An unconstrained structural model was first compared with a model in which the structural paths of interest were constrained to equality across gender groups. Group differences were evaluated using a chi-square difference test together with changes in model fit indices.

Finally, the indirect effects were estimated separately for girls and boys within the multi-group SEM, and their difference was evaluated using bias-corrected bootstrap confidence intervals. The difference was considered statistically significant when the 95% confidence interval did not include zero.

## 3 Results

### 3.1 Attrition analysis

Attrition analyses were conducted to examine potential differences between participants who completed all three waves (completers) and those who were not retained through all three waves (non-completers). Independent-samples t-tests indicated no significant differences between completers and non-completers in age (*t* = 1.103, *p* = 0.273), family functioning (*t* = 0.070, *p* = 0.944), future-oriented coping (*t* = 0.721, *p* = 0.474), or psychological well-being at T1 (*t* = 0.395, *p* = 0.694) and T2 (*t* = 0.236, *p* = 0.814). Chi-square tests revealed no significant differences between completers and non-completers in gender (χ^2^(1) = 0.013, *p* = 0.908), father’s education (χ^2^(3) = 3.236, *p* = 0.357), or mother’s education (χ^2^(3) = 3.081, *p* = 0.379). These results provided no evidence that completers and non-completers differed on the assessed demographic or study variables. Nevertheless, as with any missing-data mechanism, the missing-at-random assumption could not be verified directly.

### 3.2 Common method Bias

Given that study variables were obtained using self-report measures, potential common method variance was evaluated separately at each measurement wave. Harman’s single-factor tests showed that the first factor accounted for 21.6% of the variance at T1, 23.7% at T2, and 26.2% at T3. The wave-specific one-factor CFA fit indices were as follows: T1, CFI = 0.691, TLI = 0.614, RMSEA = 0.126, 90% CI [0.120, 0.133], SRMR = 0.102; T2, CFI = 0.814, TLI = 0.764, RMSEA = 0.096, 90% CI [0.089, 0.105], SRMR = 0.071; and T3, CFI = 0.850, TLI = 0.809, RMSEA = 0.068, 90% CI [0.064, 0.081], SRMR = 0.054. Because only psychological well-being was assessed at T3, the T3 one-factor diagnostic was less informative as a cross-construct common-method assessment than the corresponding T1 and T2 analyses. Overall, the wave-specific diagnostics did not support a dominant single-factor explanation; however, common method variance cannot be ruled out.

### 3.3 Descriptive statistics and correlations

Descriptive statistics and bivariate correlations among the study variables are presented in Table 2. Family functioning was positively associated with future-oriented coping and psychological well-being across measurement occasions. Future-oriented coping was also positively associated with psychological well-being at all assessment waves. Psychological well-being demonstrated significant positive stability over time, with scores at earlier waves being positively associated with scores at subsequent waves. In addition, perceived family SES showed small but significant positive associations with family functioning, future-oriented coping, and psychological well-being. Overall, the pattern of correlations was consistent with the hypothesized associations among the study variables.

**Table 2:** Descriptive statistics and intercorrelations among study variables.

Variable	M	SD	1	2	3	4	5	6	7	8	9
1. Age	13.02	0.82									
2. Gender	–	–	0.015								
3. Perceived family SES	–	–	−0.036	0.040							
4. Father education	–	–	0.010	−0.018	−0.018						
5. Mother education	–	–	−0.023	0.014	0.009	0.027					
6. FF (Time 1)	3.22	0.67	0.005	0.030	0.183**	0.061*	0.087**				
7. FOC (Time 2)	3.20	0.74	0.009	−0.016	0.116**	0.006	0.069**	0.311**			
8. PWB (Time 1)	3.21	0.64	−0.024	0.013	0.126**	−0.027	0.023	0.253**	0.213**		
9. PWB (Time 2)	3.19	0.66	−0.012	0.015	0.103**	0.008	0.061*	0.286**	0.321**	0.347**	
10. PWB (Time 3)	3.23	0.65	0.001	0.019	0.152**	0.034	0.074**	0.450**	0.466**	0.262**	0.483**

*p < 0.05. *^*^p < 0.01. Gender: 1 = girl, 2 = boy; Perceived family SES: 1 = Low, 2 = Middle, 3 = High; Father and mother education: 1 = Middle school or below, 2 = High school, 3 = College, 4 = Bachelor or above. SES, Socioeconomic status; FF, Family functioning; FOC, Future-oriented coping; PWB, Psychological well-being.

### 3.4 Longitudinal mediation model (future-oriented coping)

A longitudinal mediation model was estimated to examine whether future-oriented coping at T2 mediated the prospective association between family functioning at T1 and psychological well-being at T3 while accounting for temporal stability in psychological well-being and relevant demographic covariates. The model fit the data well, χ^2^(354) = 458.15, *p* < 0.001, χ^2^/df = 1.29, CFI = 0.988, TLI = 0.986, RMSEA = 0.014 (90% CI [0.010, 0.018]), and SRMR = 0.030.

As shown in Figure 2 and Table 3, family functioning at T1 was positively associated with future-oriented coping at T2 (*β* = 0.448, *p* < 0.001) and psychological well-being at T2 (*β* = 0.266, *p* < 0.001). Family functioning at T1 also remained positively associated with psychological well-being at T3 after controlling for prior levels of psychological well-being (*β* = 0.257, *p* < 0.001). In addition, psychological well-being at T1 was positively associated with future-oriented coping at T2 (*β* = 0.152, *p* < 0.001), and psychological well-being demonstrated significant temporal stability across waves, with T1 psychological well-being positively predicting T2 psychological well-being (*β* = 0.347, *p* < 0.001) and T2 psychological well-being positively predicting T3 psychological well-being (*β* = 0.301, *p* < 0.001).

**Figure 2:**
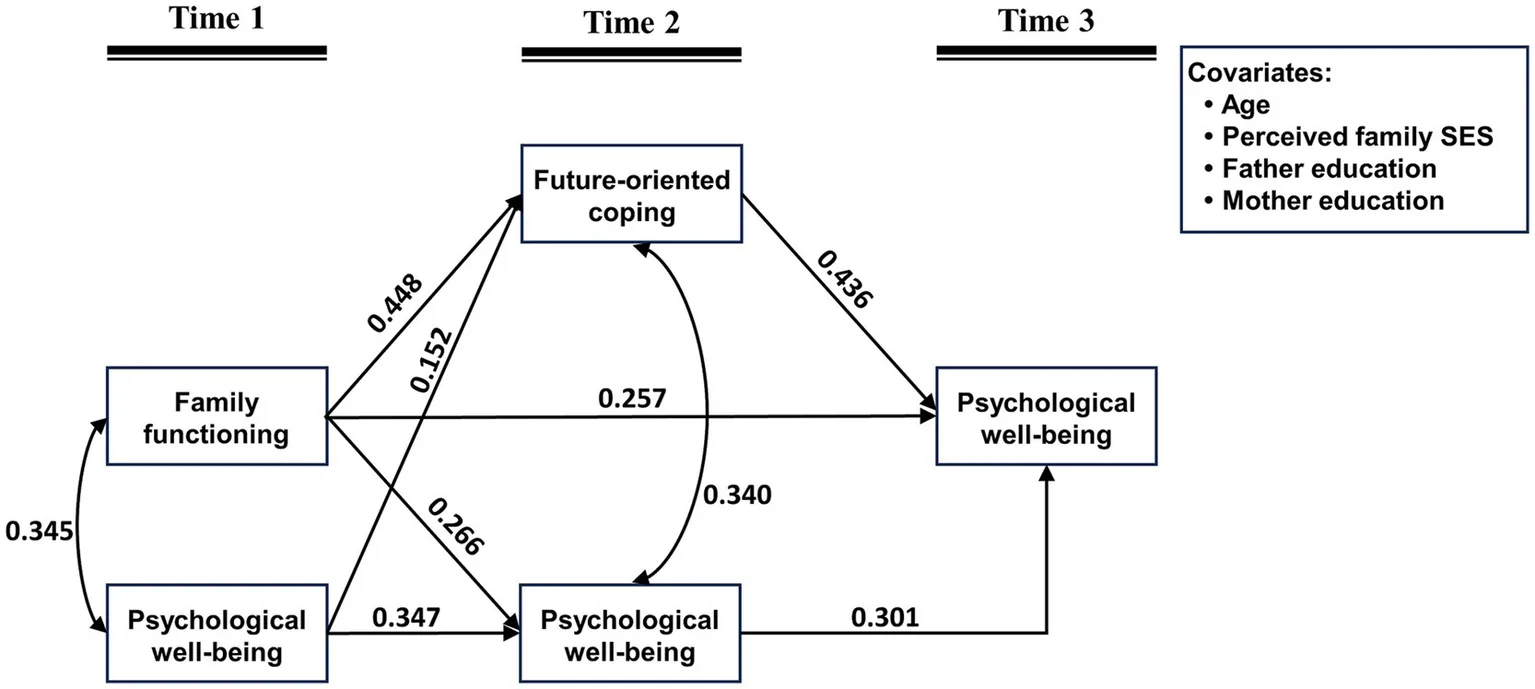
Standardized path coefficients for the longitudinal mediation model.

**Table 3:** Standardized structural paths and indirect effects in the longitudinal mediation model.

Path	B [95% CI]	SE	*β* [95% CI]	*p*-value
PWB1 → PWB2	0.403 [0.321, 0.489]	0.043	0.347 [0.283, 0.412]	< 0.001
PWB2 → PWB3	0.437 [0.326, 0.550]	0.057	0.301 [0.227, 0.374]	< 0.001
FF → FOC	0.527 [0.409, 0.658]	0.064	0.448 [0.361, 0.535]	< 0.001
PWB1 → FOC	0.178 [0.085, 0.273]	0.048	0.152 [0.073, 0.231]	< 0.001
FF → PWB2	0.308 [0.219, 0.394]	0.044	0.266 [0.196, 0.335]	< 0.001
FF → PWB3	0.433 [0.295, 0.575]	0.070	0.257 [0.177, 0.336]	< 0.001
FOC → PWB3	0.625 [0.469, 0.817]	0.089	0.436 [0.339, 0.532]	< 0.001
Indirect effect: FF → FOC → PWB3	0.330 [0.224, 0.474]	0.064	0.195 [0.132, 0.258]	< 0.001
Total effect: FF → PWB3	0.763 [0.615, 0.917]	0.078	0.452 [0.386, 0.518]	< 0.001

FF, Family function; FOC, Future-oriented coping; PWB, Psychological well-being; B, unstandardized coefficient; SE, bootstrap standard error; *β*, standardized coefficient; CI, confidence interval.

Consistent with the hypothesized mediation model, future-oriented coping at T2 was positively associated with psychological well-being at T3 after accounting for prior psychological well-being and covariates (*β* = 0.436, *p* < 0.001). Bootstrapping analyses (5,000 resamples) further indicated a significant indirect effect of family functioning at T1 on psychological well-being at T3 through future-oriented coping at T2 (*β* = 0.195, 95% CI [0.132, 0.258]). The total prospective association between family functioning at T1 and psychological well-being at T3 was also significant (*β* = 0.452, 95% CI [0.386, 0.518]). The significant indirect effect suggests that future-oriented coping partially accounted for the prospective association between family functioning and subsequent psychological well-being.

### 3.5 Gender differences in the longitudinal model

Measurement invariance testing supported configural, metric, and scalar invariance across gender, indicating that comparisons of structural relations between girls and boys were appropriate. A multi-group SEM was subsequently conducted to examine whether the longitudinal associations among family functioning, future-oriented coping, and psychological well-being differed across gender. The unconstrained multi-group model demonstrated a good fit to the data, χ^2^(708) = 818.78, *p* = 0.002, CFI = 0.987, TLI = 0.985, RMSEA = 0.014 (90% CI [0.009, 0.019]), and SRMR = 0.035.

To evaluate gender differences in structural pathways, a constrained model in which all structural paths were fixed to be equal across groups was compared with the unconstrained model. The chi-square difference test indicated that constraining the structural paths significantly worsened model fit, Δχ^2^(7) = 21.24, *p* = 0.003, suggesting that at least one longitudinal association differed between girls and boys.

As shown in Figure 3 and Table 4, family functioning at T1 positively predicted future-oriented coping at T2 in both girls (*β* = 0.543, *p* < 0.001) and boys (*β* = 0.349, *p* < 0.001), although the standardized coefficient was numerically larger among girls. Similarly, future-oriented coping at T2 positively predicted psychological well-being at T3 in both girls (*β* = 0.546, *p* < 0.001) and boys (*β* = 0.302, *p* < 0.001), with a numerically larger standardized coefficient among girls. In contrast, the standardized coefficient for the direct association between family functioning at T1 and psychological well-being at T3 was numerically larger among boys (*β* = 0.364, *p* < 0.001) than among girls (*β* = 0.153, *p* = 0.024). The autoregressive paths linking psychological well-being across waves were significant in both gender groups and showed similar standardized coefficients. Overall, the multi-group analysis indicated that structural relations varied across gender groups. Figure 4 visually summarizes the standardized path and effect estimates for girls and boys.

**Figure 3:**
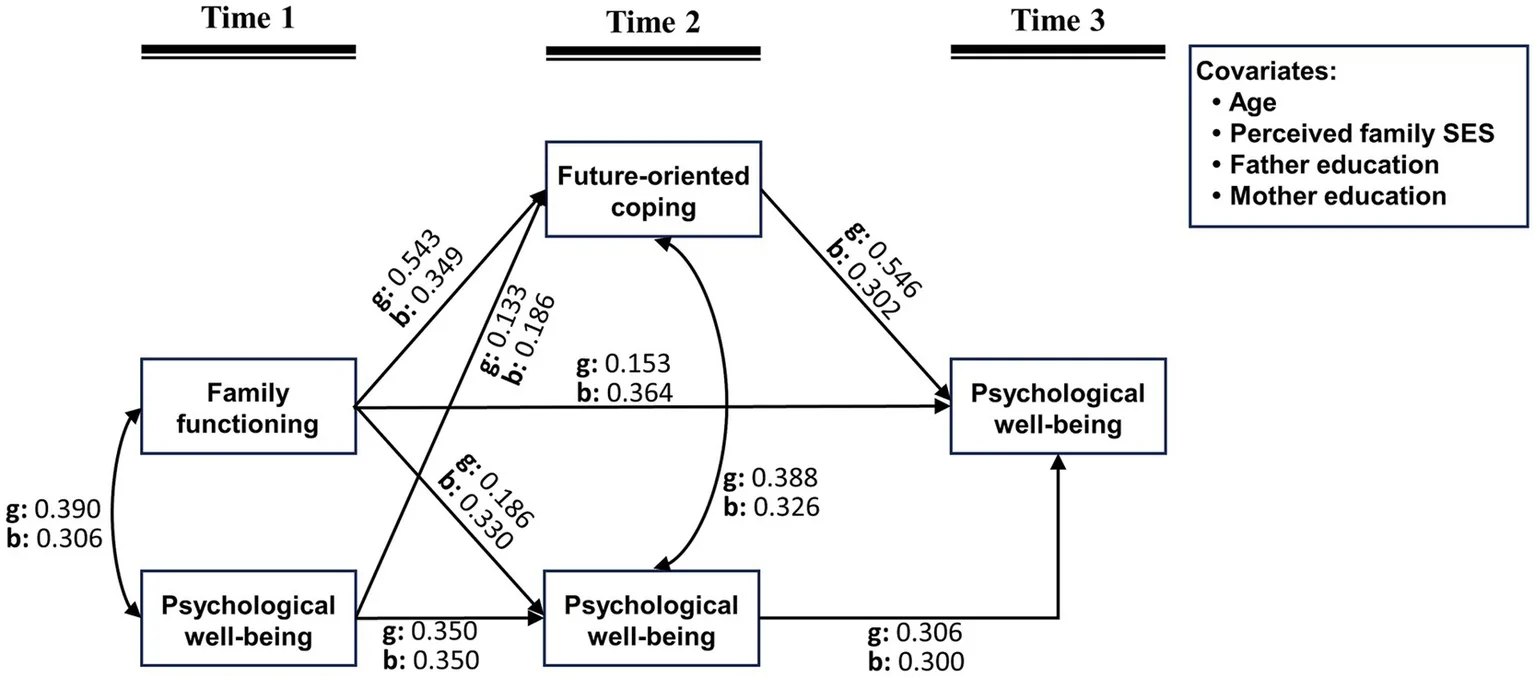
Multi-group longitudinal structural equation model showing standardized path coefficients for girls (g) and boys (b).

**Table 4:** Multi-group structural path estimates for the longitudinal model across gender.

Path	Girls				Boys			
	B [95% CI]	SE	*β* [95% CI]	*p*-value	B [95% CI]	SE	*β* [95% CI]	*p*-value
PWB1 → PWB2	0.395 [0.269, 0.523]	0.066	0.350 [0.247, 0.454]	< 0.001	0.420 [0.306, 0.538]	0.059	0.350 [0.265, 0.435]	< 0.001
PWB2 → PWB3	0.512 [0.326, 0.704]	0.096	0.306 [0.202, 0.411]	< 0.001	0.389 [0.239, 0.543]	0.078	0.300 [0.187, 0.412]	< 0.001
FF → FOC	0.691 [0.499, 0.904]	0.106	0.543 [0.424, 0.662]	< 0.001	0.391 [0.241, 0.578]	0.084	0.349 [0.219, 0.480]	< 0.001
PWB1 → FOC	0.170 [0.030, 0.308]	0.071	0.133 [0.024, 0.243]	0.017	0.208 [0.067, 0.356]	0.074	0.186 [0.061, 0.312]	0.005
FF → PWB2	0.210 [0.080, 0.342]	0.066	0.186 [0.073, 0.299]	0.002	0.396 [0.279, 0.512]	0.060	0.330 [0.243, 0.417]	< 0.001
FF → PWB3	0.288 [0.007, 0.514]	0.127	0.153 [0.014, 0.292]	0.024	0.569 [0.385, 0.757]	0.096	0.364 [0.261, 0.468]	< 0.001
FOC → PWB3	0.809 [0.533, 1.145]	0.165	0.546 [0.387, 0.705]	< 0.001	0.421 [0.229, 0.685]	0.116	0.302 [0.159, 0.444]	< 0.001

FF, Family functioning; FOC, Future-oriented coping; PWB, Psychological well-being; B, unstandardized coefficient; SE, standard error; *β*, standardized coefficient; CI, confidence interval.

**Figure 4:**
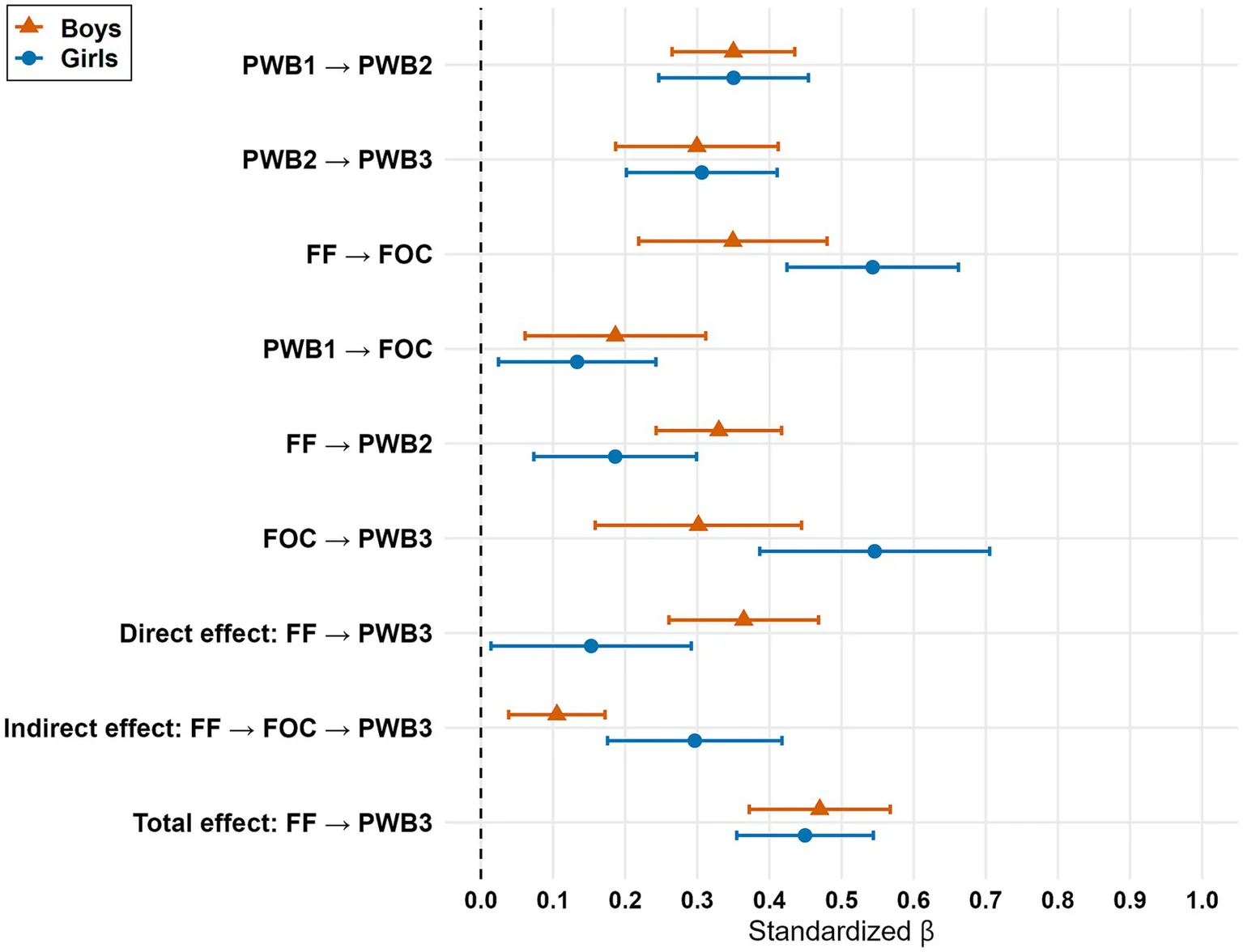
Forest plot of standardized path and effect estimates with 95% confidence intervals for girls and boys.

In addition to the structural-path comparisons, the gender difference in the indirect longitudinal association between family functioning at T1 and psychological well-being at T3 through future-oriented coping at T2 was evaluated using bias-corrected bootstrap analyses (5,000 resamples) within the multi-group SEM framework. The results are presented in Table 5.

**Table 5:** Gender differences in the indirect longitudinal association between family functioning and psychological well-being through future-oriented coping.

Effect	B [95% CI]	SE	*β* [95% CI]	*p*-value
Indirect effect (Girls)	0.559 [0.335, 0.881]	0.155	0.297 [0.176, 0.417]	< 0.001
Indirect effect (Boys)	0.164 [0.081, 0.322]	0.061	0.105 [0.039, 0.172]	0.007
Difference (Girls − Boys)	0.394 [0.122, 0.750]	0.166	0.191 [0.054, 0.329]	0.017

B, unstandardized indirect effect; SE, bootstrap standard error; *β*, standardized indirect effect; CI, bias-corrected bootstrap confidence interval.

The indirect effect of family functioning at T1 on psychological well-being at T3 through future-oriented coping at T2 was significant for both girls (B = 0.559, 95% CI [0.335, 0.881], *β* = 0.297, 95% CI [0.176, 0.417], *p* < 0.001) and boys (B = 0.164, 95% CI [0.081, 0.322], *β* = 0.105, 95% CI [0.039, 0.172], *p* = 0.007). These findings indicate that future-oriented coping partially accounted for the prospective association between family functioning and subsequent psychological well-being in both gender groups.

The difference between the indirect effects was statistically significant (ΔB = 0.394, 95% CI [0.122, 0.750], *β* = 0.191, 95% CI [0.054, 0.329], *p* = 0.017), indicating that the indirect longitudinal association was stronger among girls than boys.

## 4 Discussion

The present three-wave prospective study examined whether future-oriented coping prospectively accounted for the association between family functioning and psychological well-being in Chinese early adolescents and whether these longitudinal pathways varied by gender. Several important findings emerged. First, more positive family functioning was prospectively associated with greater future-oriented coping and higher psychological well-being, even after prior levels of psychological well-being were taken into account. Second, greater future-oriented coping was associated with higher subsequent psychological well-being. Third, future-oriented coping partially accounted for the longitudinal association between family functioning and later psychological well-being. Finally, the structural pathways differed by gender, with the indirect association being stronger among girls. Taken together, these findings extend prior research by supporting future-oriented coping as one potential temporally ordered pathway linking family functioning to adolescent psychological well-being.

### 4.1 Family functioning and psychological well-being

The positive association between family functioning and psychological well-being is consistent with longitudinal evidence showing that healthier family environments are linked to a broad range of adolescent well-being outcomes, including life satisfaction, self-esteem, purpose in life, and lower psychological distress (Shek, 1998, 2005). Within the Chinese sociocultural context examined in this study, family cohesion, interdependence, and relational harmony may be salient aspects of adolescents’ family experiences. Supportive family functioning may therefore be associated with emotional security, stable expectations, and a sense of belonging that supports positive psychological development (Lin et al., 2014). The present findings suggest that these family-related processes are relevant not only to concurrent well-being but also to subsequent well-being across time.

The longitudinal nature of the present study strengthens this interpretation. By controlling for prior psychological well-being, the observed association between family functioning and later well-being suggests that family experiences were associated with subsequent psychological well-being above and beyond earlier levels of well-being. This pattern is consistent with the view that family functioning remains an important developmental context during early adolescence, a period marked by increasing autonomy and major school and social transitions (Eccles, 1999). Supportive family environments may therefore serve as an important relational foundation for adolescent adjustment during this stage.

### 4.2 Family functioning and future-oriented coping

Better family functioning was prospectively associated with greater future-oriented coping, suggesting that adolescents who perceived their families as more supportive and well organized reported stronger tendencies toward anticipatory, planned, and resource-building coping. This finding is consistent with previous work showing that family functioning is linked to adolescents’ coping strategies and broader adjustment, including studies indicating that inadequate family functioning is associated with more maladaptive coping, whereas positive family processes are associated with more adaptive regulation and resilience (Francisco et al., 2016). It is also consistent with the possibility that communication, emotional support, modeling, and opportunities for guided problem solving are among the family processes that contribute to adolescents’ coping resources.

In early adolescence, future-oriented coping may be especially responsive to the family context because adolescents are beginning to face more complex academic and social demands while still relying heavily on family guidance. Supportive families may help adolescents develop a greater sense of preparedness for future challenges, which may in turn foster planning and proactive resource management. In this sense, family functioning may be associated with adolescents’ willingness and ability to think ahead, prepare for difficulties, and organize resources to meet anticipated demands.

### 4.3 Future-oriented coping as a longitudinal mediator

A central finding of this study was that future-oriented coping partially accounted for the longitudinal association between family functioning and psychological well-being. Adolescents who reported more positive family functioning subsequently reported greater future-oriented coping, which in turn was associated with higher levels of later psychological well-being. The direction of this pattern is broadly consistent with three-wave research in New Zealand in which family connectedness at T1 was associated with adaptive and maladaptive coping at T2, which were in turn associated with health indicators at T3 (Gervais and Jose, 2020), as well as with longitudinal evidence linking future orientation, subsequent coping, and later well-being (Chua et al., 2015). It is also consistent in direction with Spanish adolescent findings linking proactive and preventive coping with higher subjective well-being and lower depression (Serrano et al., 2021). Although differences in the constructs assessed, outcomes, follow-up intervals, and statistical adjustment limit direct comparison of effect magnitudes, the overall direction of the present findings is broadly consistent with evidence from other adolescent populations.

The present findings extend this literature in two ways. First, they suggest that future-oriented coping may represent one pathway through which family functioning is associated with later psychological well-being. Second, this indirect association was observed in a prospective model that controlled for prior well-being, providing clearer evidence of temporal ordering than is available from cross-sectional research. The significant direct association between family functioning and later psychological well-being, however, indicates that future-oriented coping did not fully account for this longitudinal association. Family functioning may be associated with adolescent well-being through multiple pathways, only one of which involves anticipatory coping.

An additional noteworthy finding was that higher initial psychological well-being was associated with greater subsequent future-oriented coping. This finding suggests that adolescents who begin with stronger psychological well-being may be better positioned to engage subsequently in planning, preparation, and future-oriented regulation. However, because future-oriented coping was assessed only at T2, the present study could not determine whether coping and well-being are reciprocally associated across time. This interpretation is compatible with longitudinal evidence showing that adolescents’ future orientation and coping skills are intertwined with later adjustment outcomes (Chua et al., 2015). It also suggests that future-oriented coping may reflect both a resource shaped by the family environment and a developmental capacity supported by earlier psychological functioning.

### 4.4 Gender differences in the longitudinal pathways

The multi-group SEM results indicated that the longitudinal pathways differed by gender, and measurement invariance supported meaningful comparison across girls and boys. Specifically, the standardized coefficients for the paths involving future-oriented coping were numerically larger among girls, whereas the standardized coefficient for the direct association between family functioning and psychological well-being was numerically larger among boys. Importantly, the indirect association through future-oriented coping was significantly stronger among girls. This pattern suggests that future-oriented coping may play a more prominent role in linking family experiences with psychological well-being for girls.

These findings are broadly consistent with evidence that girls often show greater engagement in planning, support-seeking, and active coping across adolescence (Flannery et al., 2018), and with longitudinal findings that family functioning is sometimes more strongly linked to adolescent adjustment among girls than boys in Chinese samples (Shek, 2005; Lin et al., 2014). At the same time, not all studies find clear gender differences in the average use of future-oriented coping. For example, Gil-Juliá et al. (2024) reported no overall sex differences in proactive and preventive coping. Taken together, this suggests that gender differences may lie less in whether adolescents use future-oriented coping at all, and more in how strongly such coping becomes connected to family experiences and later well-being.

The numerically larger standardized coefficient for the direct association between family functioning and psychological well-being among boys may indicate that supportive family environments are beneficial for boys through more direct or generalized pathways, whereas girls’ well-being may be more closely tied to coping-related processes. One possible interpretation is that gender-related socialization experiences or differences in the salience of family relationships may contribute to how family support becomes associated with anticipatory coping. This explanation remains tentative because these processes were not measured directly, and other developmental or contextual factors may also account for the observed group difference. This interpretation is also consistent with studies showing that gender-specific indirect effects can emerge even when mean differences in coping are limited (Martínez et al., 2019). More broadly, the present pattern suggests that gender should be considered not only as a descriptive demographic variable, but also as a moderator of how family experiences are translated into coping and well-being across development.

### 4.5 Practical implications

The present findings suggest several practical implications for promoting adolescent psychological well-being. First, the findings point to overall family functioning as a potentially relevant domain for well-being promotion during early adolescence. Family communication, emotional support, and positive parent–adolescent relationships may be candidate components for evaluation in family-focused programs. Second, the prospective association between future-oriented coping and psychological well-being suggests that planning, goal setting, problem anticipation, and proactive coping may be useful components to evaluate in school- or community-based programs. Intervention studies are needed to determine whether strengthening these resources produces improvements in psychological well-being. Third, the stronger indirect association observed among girls indicates that intervention research should examine whether the relevance or effectiveness of coping-focused components varies across gender groups, rather than assuming that different approaches are necessarily required. In educational contexts characterized by substantial academic demands and future-oriented expectations, supporting adolescents’ capacity to anticipate and prepare for future challenges may represent a useful component of broader well-being promotion initiatives.

### 4.6 Limitations and future directions

Several limitations should be acknowledged. First, the study was observational. Although the variables were assessed in the hypothesized temporal sequence and prior levels of psychological well-being were controlled, the findings do not support causal conclusions. Unmeasured third variables and alternative developmental processes may also contribute to the observed associations. Second, family functioning was assessed only at T1 and future-oriented coping only at T2. Consequently, the present design could not distinguish stable between-person differences from within-person changes or examine reciprocal dynamics across all three study constructs. Future-oriented coping was also examined as an overall construct; although its proactive and preventive components may show distinct associations with family functioning and psychological well-being, component-specific pathways were not examined in the present study. The study also focused exclusively on early adolescents and employed three-month intervals between measurement occasions, making it unclear whether the observed associations operate similarly across later developmental stages or longer periods.

Third, all variables were assessed using adolescent self-report measures. Although adolescents are appropriate informants regarding their perceptions of family functioning, coping, and psychological well-being, common method variance, social desirability, and shared reporting biases cannot be ruled out. The wave-specific diagnostic analyses did not support a dominant single-factor explanation, but they could not eliminate these concerns.

Fourth, participants were recruited from public secondary schools in a single city in northeastern China. Cultural, regional, and educational characteristics may therefore limit the generalizability of the findings. Finally, gender was operationalized using the binary categories of girls and boys, precluding examination of potentially distinct experiences among gender-diverse adolescents.

Future research should repeatedly assess family functioning, future-oriented coping, and psychological well-being across longer developmental periods and incorporate multiple informants or methods. Repeated assessment of all three constructs would permit examination of reciprocal processes and could support approaches such as random intercept cross-lagged panel models to separate within-person from between-person processes. Future studies should also examine proactive and preventive coping separately to determine whether these components show distinct associations with family functioning and psychological well-being. Such research could further clarify whether the observed associations persist across middle and late adolescence while reducing reliance on shared self-report data.

Future studies could also examine additional individual and relational processes, such as emotion regulation, resilience, and social support, alongside future-oriented coping. Contextual factors such as academic stress, school climate, and culturally relevant family values may help clarify when and for whom the observed associations are stronger or weaker. More inclusive gender measures should be used where feasible. Finally, intervention research is needed to determine whether strengthening future-oriented coping improves psychological well-being and whether the effects of coping-focused programs vary across gender groups.

## 5 Conclusion

This three-wave prospective study found that more positive family functioning was associated with later psychological well-being both directly and indirectly through future-oriented coping in Chinese early adolescents, after accounting for prior psychological well-being. The indirect association was stronger among girls than boys. These findings support future-oriented coping as one potential developmental pathway through which family experiences are linked to subsequent well-being and highlight the value of longitudinal, culturally contextualized research on family and coping processes during early adolescence.

## Data Availability

The raw data supporting the conclusions of this article will be made available by the authors, without undue reservation.
